# Ameliorative Effect of *Vernonia amygdalina* Plant Extract on Heavy Metal-Induced Liver and Kidney Dysfunction in Rats

**DOI:** 10.1155/2020/2976905

**Published:** 2020-06-24

**Authors:** Precious Barnes, Joshua Kwame Yeboah, Wilson Gbedema, Roland Osei Saahene, Benjamin Amoani

**Affiliations:** ^1^Department of Physician Assistantship, School of Allied Health Sciences, College of Health and Allied Sciences, University of Cape Coast, Cape Coast, Ghana; ^2^Department of Biomedical Sciences, School of Allied Health Sciences, College of Health and Allied Sciences, University of Cape Coast, Cape Coast, Ghana; ^3^Department of Microbiology and Immunology, School of Medical Sciences, University of Cape Coast, Cape Coast, Ghana

## Abstract

Heavy metal toxicity contributes to liver and kidney dysfunction and damage through oxidative stress mechanisms; however, from previous studies, extracts from the *Vernonia amygdalina* plant have shown to possess potent antioxidant properties. This study was aimed at uncovering the potential ameliorative effects of ethanolic extract from *Vernonia amygdalina* plant in heavy metal toxicity-induced liver and kidney dysfunction. For this study, 44 Sprague Dawley rats were divided into three groups. The control group received a basal diet and water only while the treatment groups received varied dosages of the heavy metals. The copper (Cu) and lead (Pb) groups had five subgroups. The Cu only and Cu recovery subgroups were administered with 16 mg/kg Cu intraperitoneally daily for 14 days, whereas the Pb only and Pb recovery subgroups were administered with 13 mg/kg Pb intraperitoneally daily for 14 days. Subsequently, the Pb only and Cu only subgroups were sacrificed. The three Pb and Cu treatment subgroups received oral graded doses (100 mg/kg, 200 mg/kg, and 300 mg/kg) of the extract for 21 days. The Cu recovery and Pb recovery subgroups were left to recover for 21 days. After histological examinations, the Pb and Cu pretreatment groups showed evidence of focal necrosis accompanied by inflammatory cell infiltrations. The serum levels of liver biomarkers AST, ALT, and GGT, as well as urea and creatinine, were significantly elevated (*P*=0.01) following copper and lead exposure. Upon posttreatment of the rats with the extract, the physiological levels of the biomarkers were restored and tissue architecture of the organs improved. Thus, the ethanolic extract of *Vernonia amygdalina* is capable of ameliorating the effects of heavy metal toxicity through potent antioxidative mechanisms.

## 1. Introduction

Heavy metals are defined as metallic elements that have a relatively high density compared to water. Since density and toxicity are interrelated, heavy metals such as arsenic can induce toxicity at a low level of exposure [1]. Heavy metals are well known for inducing oxidative stress. The mechanisms they apply in inducing oxidative stress include the production of reactive oxygen species (ROS), depletion of intracellular antioxidant stores and free radical scavengers, and inhibitions of antioxidative enzymes [2, 3]. The various dysfunctions displayed by cells under oxidative stress are due to damages caused by ROS to lipids, proteins, and DNA [4].

*Vernonia amygdalina* (VA) is a small shrub that predominantly grows in tropical Africa with dark green leaves and rough barks. Its bitter taste has made it to be fondly called “bitter leaf” and more importantly, it has gained wide application in the treatment of amoebic dysentery and gastrointestinal disorders and has antimicrobial, antioxidant, anti-nephrotoxic, hepatoprotective, and antiparasitic properties. Human exposure to heavy metals is almost inevitable due to their ubiquitous nature, and some are non-biodegradable, especially the nonessential heavy metals. Although there is information on the preventive effect of VA against heavy metal toxicity caused by cyanide and cadmium, much has not been done on metals like lead and copper which are more common and easy to be exposed to.

This study is aimed at bridging this gap in knowledge by providing information on the preventive effects of *V*. *amygdalina* to attenuate the toxic effects produced by excessive exposure to lead and copper by treating with ethanolic extract of *Vernonia amygdalina*.

## 2. Materials and Methods

### 2.1. Study Site

The project was undertaken in the biomedical science laboratory of the school of Allied Health Sciences, University of Cape Coast, Ghana.

### 2.2. Maintenance of Animals

A total of 44 experimental rats of about 140–180 g of both sexes were obtained from Noguchi Memorial Institute of Medical Research, University of Ghana, Legon, Ghana, and kept at the Animal House of the School of Biological Sciences, University of Cape of Coast, Ghana. They were housed in groups of six (6) in wooden cages, with wood shavings as a bed, in a well-ventilated room at room temperature and fed with normal commercial pellet diet and water. Animal Research Review Panel and Animal Welfare Unit regulations of temperature and lighting systems were maintained with a room temperature of 20–26°C as well as regular light cycles of 12 hours light/dark. All methods and protocols used in the study were observed following established public health guidelines “Guide for Care and Use of Laboratory Animals.”

### 2.3. Drugs and Chemicals

The following chemicals and drugs were used in the study: lead (II) acetate which was obtained from PARK Scientific Limited, Northampton, UK. Copper (II) sulfate pentahydrate was obtained from VWR International, Leuven, Belgium.

### 2.4. Preparation of Leaf Extracts

The *Vernonia amygdalina* leaves were collected from the Botanical Garden of the School of Biological Sciences, University of Cape Coast (UCC), Cape Coast, Central Region of Ghana. The leaves were subsequently identified by a botanist at UCC and deposited in the School's herbarium, with the accession number CE 001. The leaves were washed thoroughly with tap water to remove any form of dirt. The leaves were then air-dried under shade for 28 days at room temperature and pulverized into powder using a blender and stored in airtight plastic containers. One hundred and twenty-five grams (125 g) of the powdered leaves were macerated in separate cold ethanol (70% ethanol, 1 : 8 w/v) and were allowed to stand to at room temperature for one week. The mixture was shaken continuously for several times for a uniform mixture and to obtain a maximum extract yield. After a week, the macerated solution was filtered using a filter paper into a beaker. The filtrate was boiled on a water bath at a temperature of 70°C to separate the ethanol from the crude drug. The crude drug extract was labelled EEVA (ethanolic extract of *Vernonia amygdalina*) and was transferred into a Petri dish and stored in a desiccator to solidify and dry the extract, after which it was kept in a refrigerator at 4°C.

### 2.5. Experimental Design

The rats were separated according to sex or gender and were fed with a basal diet for a period of one (1) month of acclimatization. After one month of acclimatization, the weight of each animal was measured in grams and grouped into groups of three (3) taking into account the sex. 4 animals were allocated to the control group, 20 animals were allocated to the copper group, and 20 animals were allocated to the lead group.

The control group was given a basal diet and water only throughout the study period. The copper (Cu) group had five subgroups; the Cu only and Cu recovery subgroups (pretreatment group) were pretreated with 16 mg/kg Cu intraperitoneally daily for 14 days; however, the Cu only subgroup was sacrificed after 14 days, and the Cu recovery subgroup was left to recover for 21 days. The three copper treatment subgroups received oral graded doses of ethanolic extract of *Vernonia amygdalina* (EEVA) (100 mg/kg, 200 mg/kg, and 300 mg/kg) for 21 days, after pretreatment as the first two subgroups. The lead (Pb) group had five subgroups; the Pb only and Pb recovery subgroups (pretreatment group) were pretreated with 13 mg/kg Pb intraperitoneally daily for 14 days; however, the Pb only subgroup was sacrificed after 14 days, and the Pb recovery subgroup was left to recover for 21 days. The three lead treatment subgroups received oral graded doses of ethanolic extract of *Vernonia amygdalina* (EEVA) (100 mg/kg, 200 mg/kg, 300 mg/kg) for 21 days, after pretreatment as the first two subgroups.

### 2.6. Assessment of Biochemical Parameters

Experimental rats were sacrificed and blood was dispensed into serum separator tubes and then analyzed for biochemical parameters. The liver AST, ALT, and GGT levels and kidney urea and creatinine levels were assayed and analyzed using a standard BS-200E Mindray chemistry Autoanalyzer, PKF Scientific Limited. The AST, ALT, and GGT were measured in U/L while urea and creatinine were measured in mmol/L and *μ*mol, respectively.

### 2.7. Histological Analysis

The rats were dissected, and the liver and kidneys were removed and observed for evidence of gross pathology. For light microscopic examination, tissue samples were immediately fixed in 10% formalin, processed using an automated tissue processor (Leica Model RM 2125), and finally embedded in paraffin wax. The tissue blocks were then cut into serial sections using the rotary microtome. The sections were deparaffinized and subsequently stained with hematoxylin and eosin (H&E). The liver and hepatic microscopic architecture of experimental rats on the H&E stained slides was histologically examined.

### 2.8. Statistical Analysis

Mini tab software (version 17.1.0. cracked) was used for the data analysis. Data were presented as mean ± SEM on line graphs and tables. To compare the biological effects of the treatment, analysis of variance (ANOVA) was used. *P* values of less than 0.05 were considered statistically significant.

## 3. Results

### 3.1. Effects of Lead-Induced Heavy Metal Toxicity in Rats

The effects of lead-induced heavy metal toxicity on liver and kidney function biomarkers were investigated by comparing the AST, ALT, GGT, urea, and creatinine levels of the control group and Pb + recovery group (Table 1). There was a positive significant association (*P* < 0.05) between the two groups with a recorded *R*-squared value of more than 95% (Table 1). This indicates that more than 95% of the increase in variation in the ALT, AST, GGT, urea, and creatinine levels may be due to the effect of the administered lead (II) acetate.

### 3.2. Effects of Copper-Induced Heavy Metal Toxicity in Rats

To ascertain the effect of the copper-induced heavy metal toxicity on the liver and kidney function, the AST, ALT, GGT, urea, and creatinine levels of the control group and Cu + recovery group were compared (Table 2). There was a positive significant association (*P* < 0.05) between the two groups with a recorded *R*-squared value of more than 95%. This indicates that more than 95% of the increase in variation in the ALT, AST, GGT, urea, and creatinine levels may be due to the effect of the administered copper (II) sulfate pentahydrate.

### 3.3. Effects of EEVA on Liver and Kidney Function Biomarkers in Rats with Lead- (Pb-) Induced Heavy Metal Toxicity

The plasma levels of AST, ALT, GGT, urea, and creatinine were significantly elevated following lead exposure. However, these levels were significantly restored to physiological levels in the Pb + 100 mg/kg, Pb + 200 mg/kg, and Pb + 300 mg/kg posttreatment groups when compared to the Pb only and Pb + recovery pretreatment groups at *P* value <0.05 (Table 3). Also, the *R*-squared values of the posttreatment groups were 95% and above. This indicates that more than 95% of the decrease in variations in the AST, ALT, GGT, urea, and creatinine levels may be due to the treatment with the EEVA.

### 3.4. Effects of EEVA on Liver and Kidney Function Biomarkers in Rats with Copper-Induced Heavy Metal Toxicity

Similarly, the plasma levels of AST, ALT, GGT, urea, and creatinine were significantly elevated following copper exposure. However, these levels were significantly restored to physiological levels in the Cu + 100 mg/kg, Cu + 200 mg/kg, and Cu + 300 mg/kg posttreatment groups when compared to the Cu only and Cu + recovery pretreatment groups at *P* value <0.05 (Table 4). An average *R*-squared value of 94% of the posttreatment groups indicates that more than 94% of the decrease in variations in the AST, ALT, GGT, urea, and creatinine levels may be due to the treatment with the EEVA.

### 3.5. Histological Effects of EEVA on the Liver and Kidney of Rats Exposed to Lead and Copper Toxicity

**Figure 1:**
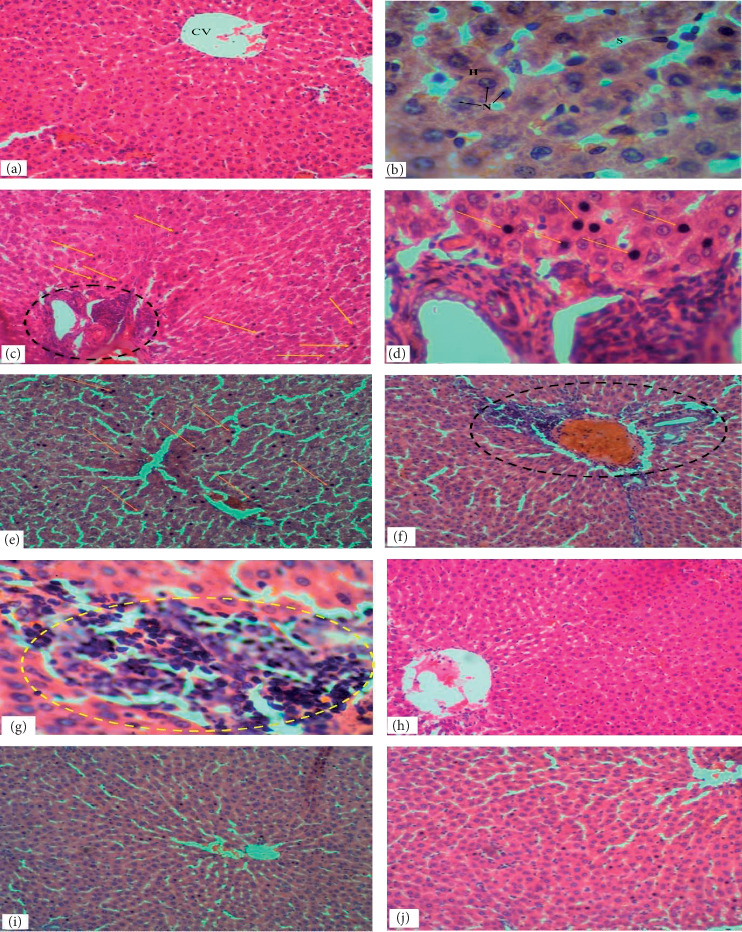
Cross sections of the liver stained with H&E. (a, b) Hepatic tissue of the control group demonstrating normal parenchymal structure, intact lamellar pattern of the hepatocytes (H) and Kupffer cells, and normal sinusoidal architecture (S), and no significant lesions were observed. (c, d) Hepatic tissue of Pb only treated rat showing evidence of focal necrotic areas accompanied with inflammatory cell infiltrations (black dotted circle) and high distributed necrosis (yellow arrows) in the liver. (e) Hepatic tissue of Cu only treated rat showing evidence of high distributed necrosis in the liver (yellow arrows). (f, g) Hepatic tissue of both Cu and Pb recovery group rats, respectively, showing evidence of focal necrotic areas accompanied with inflammatory cell infiltrations (black and yellow dotted circles) and mild disseminated necrosis in the liver. (h–j) Hepatic tissue of rat treated with heavy metals + 100 mg, 200 mg, and 300 mg of EEVA, respectively, showing improvement in parenchymal structure and normal hepatic histoarchitecture (mic. mag. (a, c, e, f, h, i) ×100; (b, d, g) ×400).

**Figure 2:**
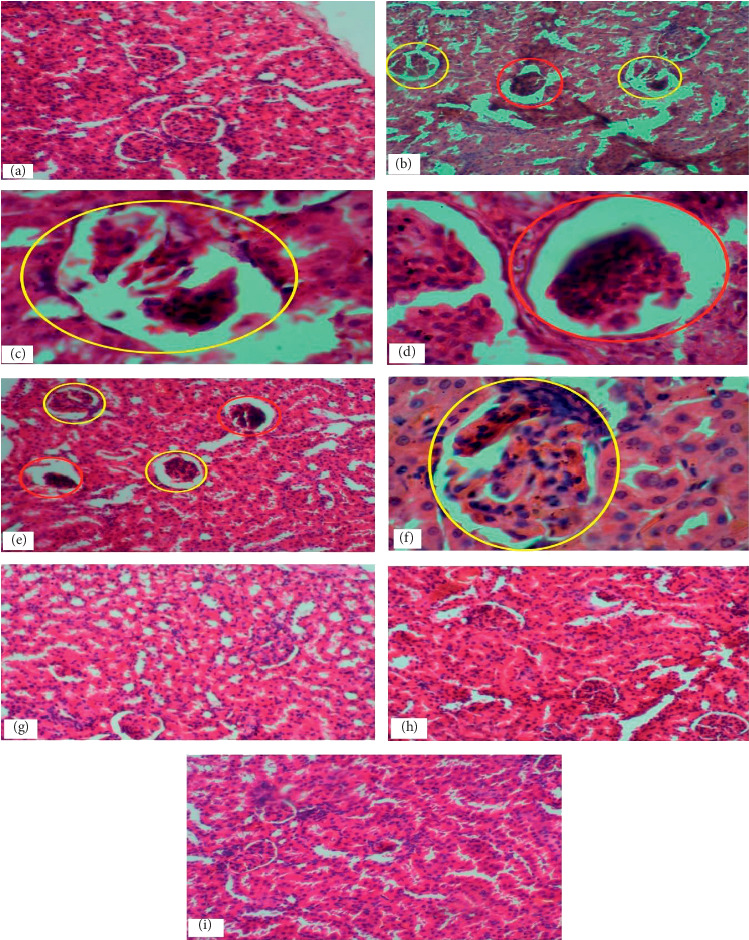
Cross sections in the cortex of the kidney stained with H&E. (a, b) Renal tissue of the control group demonstrating normal appearance of glomerular tuft, urinary space, Bowman's capsule, proximal tubules, and distal tubules with their nuclei. (b, c) Renal tissue of Cu only treated rat showing disruption of Bowman's capsule accompanied with acute cortical necrosis (red circle) and shrinking and degeneration of glomeruli (yellow circle). (d) Renal tissue of Pb only treated rat showing evidence of disrupted Bowman's capsule accompanied with acute cortical necrosis. (e, f) Renal tissue of both Cu and Pb recovery group rats, respectively, showing evidence of milder form of shrinking and degeneration of glomeruli (yellow circle) and severe disruption of Bowman's capsule accompanied with acute cortical necrosis. (g–i) Renal tissue of rat treated with heavy metals + 100 mg, 200 mg, and 300 mg of EEVA, respectively, showing improvement in glomerular tuft and renal tubules, with normal Bowman's capsule histoarchitecture (mic. mag. (a, b, e, g, h, i) ×100; (c, d, f) ×400).

The hepatic parenchyma of rats in the control group revealed evidence of apparently normal liver histoarchitecture characterized by normal parenchymal structure, intact lamellar pattern of the hepatocytes and Kupffer cells, and normal sinusoidal architecture, and no significant lesions were observed (Figure 1(a)). Also, the kidney of rats in the control group showed normal renal histoarchitecture characterized by normal glomerulus structure, capsular space, normal macula densa, and normal tubular architecture (Figure 2(a)).

Pretreatment with lead in the Pb only subgroup caused severe liver and kidney damage with evidence of focal necrotic areas accompanied with inflammatory cell infiltrations and high distributed necrosis in the liver (Figures 1(c) and 1(d)) and also evidence of disrupted Bowman's capsule accompanied with acute cortical necrosis in the kidney (Figure 2(d)). The liver and kidney histoarchitecture of the Pb + recovery subgroup also showed evidence of focal necrotic areas accompanied with inflammatory cell infiltrations and mild disseminated necrosis in the liver (Figure 1(g)) and severe disruption of Bowman's capsule and a mild acute cortical necrosis in the kidney (Figure 2(f)). Pretreatment with copper also caused similar liver and kidney damage with evidence of high distributed necrosis in the liver (Figure 1(e)) and disruption of Bowman's capsule with acute cortical necrosis and shrinking of glomeruli in the kidney (Figures 2(b) and 2(c)) of the Cu only subgroup, while the Cu + recovery subgroup showed evidence of mild disseminated necrosis and mild acute cortical necrosis with a milder form of glomeruli shrinkage in the liver and kidney, respectively (Figures 1(f) and 2(e)).

However, after the administration of the EEVA, all the treatment subgroups of both lead and copper groups showed ameliorative effects against the heavy metal-induced disruption in liver and kidney histoarchitecture (Figures 1(h)–1(j) and 2(g)–2(i)).

## 4. Discussion

Based on the indices assayed in this study, the overall findings showed that graded doses of ethanolic extract of *Vernonia amygdalina* (EEVA) administration restored the liver and kidney function biomarkers to normal physiological levels. Assaying of the blood levels of AST, ALT, and GGT as important biomarkers of liver function and blood levels of urea and creatinine as important biomarkers of kidney function is of high clinical relevance [5]. AST, ALT, and GGT are enzymes located in the liver cells and are usually released into the blood plasma following a liver injury or damage. However, AST is widely used to assess liver damage since it is located in both mitochondria and cytoplasm of the cells, unlike ALT which is found only in the cytoplasm.

In this study, there was a significant increase (*P* < 0.05) in the serum levels of AST, ALT, GGT, urea, and creatinine in the recovery subgroups of both copper and lead (Tables 1 and 2) when compared to the control, similar to a work by Alwaleedi [6], where the levels of AST and ALT increased significantly after administering lead acetate. These results also agree with previous studies that reported an increase in AST and ALT levels after lead administration which was caused by acute hepatitis, jaundice, and liver cirrhosis [7, 8]. Also, the increase in kidney biomarkers may be due to acute nephropathy which is characterized by impaired tubular transport and acute cortical necrosis [9]. Lead is known to have hepatotoxic and nephrotoxic effect in rats resulting in liver and kidney cell damage, which causes an increase in serum levels of AST, ALT, GGT, urea, and creatinine. There have been many reported studies that show that copper toxicity is well correlated to renal and hepatic dysfunction [10]. Copper injection in rats induces nephrotoxicity with accompanying renal dysfunction which may indicate that copper mediates in oxidative-induced renal dysfunction [11]. An increase in the levels of these liver and kidney function biomarkers might be resulting from a lead (Pb) and copper toxicity which is capable of generating free radicals that trigger a cascade of events leading to destructive alteration of the liver and kidney, respectively.

After administering the ethanolic extract of *Vernonia amygdalina* (EEVA) for 21 days, there was a significant reduction (*P* < 0.05) in the AST, ALT, GGT, urea, and creatinine levels to normal physiological levels in all the 100 mg/kg, 200 mg/kg, and 300 mg/kg EEVA treatment subgroups of both copper and lead groups (Tables 3 and 4), when compared to the recovery subgroups of both lead and copper groups. This indicates a significant positive association between the EEVA treatment subgroups of both lead and copper and the recovery subgroups of both lead and copper at an average *R*-squared value of more than 95%. This means that more than 95% of the reduction in variation of the AST, ALT, GGT, urea, and creatinine levels may be due to the administration of the EEVA. Hence, there is a significant positive association/correlation between heavy metal toxicity and treatment with *Vernonia amygdalina.* This is similar to a work by Asante et al. [12], where the levels of ALT, AST, and ALP were significantly reduced with the administration of *Vernonia amygdalina* extract in a high dose of 300 mg as it possesses antioxidant and antihyperglycemic effect.

Histological examinations of the liver of the rats in the control group revealed evidence of apparently normal liver histoarchitecture characterized by normal parenchymal structure, intact lamellar pattern of the hepatocytes and Kupffer cells, and normal sinusoidal architecture, and no significant lesions were observed, similar to a work by Imafidon et al. [13]. Likewise, the kidney of the control group showed normal renal histoarchitecture characterized by normal glomerulus structure, capsular space, normal macula densa, and normal tubular architecture.

For the lead group, the Pb only subgroup showed evidence of focal necrotic areas accompanied with inflammatory cell infiltrations and high distributed necrosis in the liver and also showed evidence of acute cortical necrosis in the kidney (Figures 1(c), 1(d), 2(b), and 2(c), respectively) which is similar to a study by Nabil [14]. These observations may be due to the lead-induced oxidative stress which may have caused cell membrane damage of hepatocytes and nephrons [6]. The aggregation of inflammatory cells may be as a result of the host immune response to combat the toxicity. However, in the examination of the liver and kidney histoarchitecture of the Pb + recovery subgroup, it showed evidence of focal necrotic areas accompanied by inflammatory cell infiltrations and mild disseminated necrosis in the liver and a mild acute cortical necrosis in the kidney (Figures 1(g) and 2(f)). The effect of the lead toxicity reduced to some extent, though insignificant, in the Pb + recovery subgroup. This may be due to the ability of the host immune defence mechanism in an attempt to eliminate the toxic lead metal.

For the copper group, similar histological observations were made. Histological examination of the liver and kidney of the Cu only subgroup revealed evidence of high distributed necrosis in the liver and acute cortical necrosis in the kidney (Figures 1(e), 2(b), and 2(c), respectively). The mechanism leading to this observation may be similar to that of the lead group. Also, the Cu + recovery group showed evidence of mild disseminated necrosis and mild acute cortical necrosis in the liver and kidney, respectively (Figures 1(f) and 2(e)).

However, after the administration of the EEVA, all the treatment subgroups of both lead and copper groups showed ameliorative effects against the heavy metal-induced disruption in liver and kidney histoarchitecture (Figures 1(h), 1(j), and 2(g)–2(i)) similar to a study by Imafidon et al. [13]. This was characterized by a few mild disseminated necroses when compared to the Pb only, Cu only, Pb recovery, and Cu recovery subgroups.

## Figures and Tables

**Table 1:** Distribution on the effect of lead-induced heavy metal toxicity in rats.

Variable	Indicator	Mean ± SD	95% CI for (lower, upper)	*R*-squared (%)	Sig
Lead group					
“Control GRP”	AST	76.0 ± 2.416	(69.199, 82.801)	94.13	0.01
	ALT	66.483 ± 0.947	(65.267, 67.7)	99.53	0.01

VRS					
“Pb + recovered”	GGT	57.067 ± 1.186	(56.185, 57.949)	99.65	0.01
	Urea	15.2 ± 1.270	(14.3, 16.1)	96.82	0.01
	Creatinine	166.33 ± 8.02	(160.91, 171.76)	98.32	0.01

Values are presented as mean ± SD with 95% CI and *R*-squared (%). *P* value <0.05 is statistically significant at a significant level of 95%.

**Table 2:** Distribution on the effect of copper-induced heavy metal toxicity in rats.

Variable	Indicator	Mean ± SD	95% CI for (lower, upper)	*R*-squared (%)	Sig
Copper group					
“Control GRP”	AST	63.62 ± 3.61	(56.60, 70.63)	90.02	0.01
	ALT	59.12 ± 2.50	(57.20, 61.04)	98.14	0.01

VRS					
“Cu + recovered”	GGT	53.433 ± 2.412	(51.82, 55.047)	98.49	0.01
	Urea	14.0 ± 1.757	(12.808, 15.192)	93.07	0.01
	Creatinine	157.50 ± 9.35	(151.25, 163.75)	97.25	0.01

Values are presented as mean ± SD with 95% CI and *R*-squared (%). *P* value <0.05 is statistically significant at a significant level of 95%.

**Table 3:** Effects of EEVA on liver and kidney function biomarkers in rats with lead- (Pb-) induced heavy metal toxicity.

Lead group	Indicators (mean ± SD)				
	AST	ALT	GGT	Urea	Creatinine
Control group	21.32 ± 10.29	31.10 ± 1.637	27.283 ± 0.688	5.233 ± 0.589	83.0 ± 2.61
Pb only	95.63 ± 4.25	80.20 ± 2.53	73.98 ± 3.71	17.967 ± 0.582	194.50 ± 7.06
Pb + recovery	76.0 ± 2.416	66.483 ± 0.947	57.067 ± 1.186	15.2 ± 1.270	166.33 ± 8.02
Pb + 100 mg/kg	39.483 ± 0.937	49.2 ± 1.430	47.367 ± 1.140	7.9833 ± 0.2137	118.33 ± 2.50
Pb + 200 mg/kg	35.283 ± 0.422	42.367 ± 2.062	36.950 ± 1.607	7.350 ± 0.409	108.5 ± 7.01
Pb + 300 mg/kg	30.483 ± 1.201	35.217 ± 0.847	31.0 ± 1.362	6.617 ± 0.299	94.92 ± 3.23

Values are presented as mean ± SD. *P* value <0.05 is statistically significant.

**Table 4:** Effects of EEVA on liver and kidney function biomarkers in rats with copper- (Cu-) induced heavy metal toxicity.

Copper group	Indicators (mean ± SD)				
	AST	ALT	GGT	Urea	Creatinine
Control group	21.32 ± 10.29	31.10 ± 1.637	27.283 ± 0.688	5.233 ± 0.589	83.0 ± 2.61
Cu only	88.97 ± 4.06	70.78 ± 6.72	67.58 ± 4.05	16.75 ± 0.734	180.67 ± 2.73
Cu + recovery	63.62 ± 3.61	59.12 ± 2.50	53.433 ± 2.412	14.0 ± 1.757	157.5 ± 9.35
Cu + 100 mg/kg	37.333 ± 0.774	46.183 ± 0.854	43.35 ± 2.36	7.717 ± 0.248	114.33 ± 3.33
Cu + 200 mg/kg	32.583 ± 1.028	36.967 ± 1.167	33.667 ± 1.213	7.0667 ± 0.2422	102.5 ± 2.66
Cu + 300 mg/kg	28.267 ± 0.680	34.1 ± 0.780	29.817 ± 0.739	6.2667 ± 0.2160	90.5 ± 1.871

Values are presented as mean ± SD. *P* value <0.05 is statistically significant.

## Data Availability

The data can be accessed from University of Cape Coast Library (http://www.ucc.edu.gh).
